# Soluble MHC class I chain-related protein A is a specific biomarker for the early detection of graft-versus-host disease

**DOI:** 10.3389/fmed.2025.1580452

**Published:** 2025-06-05

**Authors:** Alexander Kupis, Nina Buchtele, Philipp Wohlfarth, Werner Rabitsch, Katarina D. Kovacevic Miljevic, Marion Mussbacher, Bernd Jilma, Christian Schoergenhofer

**Affiliations:** ^1^Department of Clinical Pharmacology, Medical University of Vienna, Vienna, Austria; ^2^Department of Internal Medicine I, Intensive Care Unit 13i2, Medical University of Vienna, Vienna, Austria; ^3^Department of Internal Medicine I, Bone Marrow Transplantation Unit, Medical University of Vienna, Vienna, Austria; ^4^Department of Pharmacology and Toxicology, Institute of Pharmaceutical Sciences, University of Graz, Graz, Austria

**Keywords:** soluble MHC class I chain-related protein A, sMICA, biomarker, graft-versus host disease, GvHD, allogeneic hematopoetic stem cell transplantation

## Abstract

**Introduction:**

MHC class I chain-related protein A (MICA) acts as a marker of cellular stress and its expression is a destruction-signal for NKG2D-expressing cytotoxic cells. Soluble MICA (sMICA) concentrations after allogeneic hematopoietic stem cell transplantation (HSCT) were associated with worse outcomes and graft-versus-host disease (GVHD). We hypothesized that (i) sMICA could be a prognostic biomarker for the development of GVHD and (ii) may act as an acute phase reactant.

**Methods:**

In this prospective study we included 48 patients undergoing HSCT and drew blood samples before conditioning (baseline), during engraftment and 100 days after HSCT. The follow-up period was 1 year for each patient. Soluble MICA and established acute phase reactants (C-reactive Protein, von Willebrand Factor) were measured by enzyme-linked immunoassay (ELISA).

**Results:**

Of the 44 patients in the final analysis, 30 (68%) developed GVHD (16 acute GVHD, 8 chronic GVHD, 6 acute and chronic GVHD). Soluble MICA concentrations at baseline and during engraftment were significantly higher in patients who developed acute or chronic GVHD (*p* = 0.017). Receiver operating characteristic (ROC) curve analysis for the baseline values showed an area under the curve of 0.78 (*p* < 0.001; 95% confidence intervals 0.64–0.91) for diagnosis of acute or chronic GVHD. Soluble MICA concentrations above 93.5 pg/mL had a specificity of 93% for the diagnosis of GVHD, while the sensitivity was only 47%.

**Discussion:**

Soluble MICA did not correlate with other acute phase reactants and remained stable during engraftment. Soluble MICA may potentially serve as a biomarker with high specificity for the prediction of GVHD.

## Introduction

Hematopoietic stem cell transplantation (HSCT) is a well-established treatment for malignant and non-malignant hematological diseases incurable by conventional treatments (1). Graft-versus-host disease (GVHD), both acute and chronic, is one of the most important complications following allogeneic HSCT and still accounts for major therapy-related morbidity and mortality (2, 3). Thus, prediction and early identification of GVHD, as well as potential adjustment of immunosuppressive therapy for GVHD is key in order to improve patient outcomes.

The MHC class I chain-related protein A (MICA) plays an important role in immunobiology. MICA transcripts have been detected in practically every tissue except the central nervous system. Its expression on the cell surface is limited to gastrointestinal epithelial cells, endothelial cells, fibroblasts and monocytes (4, 5). In the setting of organ transplantation, the ubiquitous expression of MICA combined with its highly polymorphic nature as an HLA-related antigen may contribute to the induction of alloreactivity (5, 6). MICA mismatches can significantly increase the risk of transplant-related complications, such as graft rejection (7). In allogeneic HSCT matching for MICA polymorphisms significantly improved outcomes and was associated with lower rates of GVHD (8).

Apart from triggering alloreactivity, MICA acts as a ligand for the natural killer group 2, member D (NKG2D) receptor which is an activating receptor predominantly expressed on cytotoxic immune cells, such as NK cells, NKT cells, *γ*:*δ* T cells, and CD8^+^ *α*:*β* T cells (9, 10). Its expression can be upregulated by genotoxic and cytotoxic stress (e.g., chemotherapy, heat shock, proliferative signals, malignant transformation, infection, oxidative stress, etc.) (10, 11). However, chronic engagement of NKG2D has been shown to mediate its own downregulation, resulting in subsequent reduced responsiveness of NK and T cells to other stimuli (12). This was observed in tumors that paradoxically express high amounts of MICA, which is subsequently shed from the cell surface by metalloproteases (13). This “soluble” MICA reduced cell surface expression of NKG2D on tumor infiltrating lymphocytes (TIL), NK cells and *γ*:*δ* T cells, ultimately resulting in an impaired responsiveness to NKG2D activation (12).

However, this mechanism may not only allow tumor cells to evade the immune response, but may also be important for the development of GVHD. Olson et al. (14) demonstrated that alloreactive donor NK cells may suppress the development of GVHD by regulating alloreactive T cells and antigen presenting cells (APC). Interestingly, in these experiments the graft-versus-leukemia (GVL) effect was retained (14). Chronic stimulation with (soluble) MICA may therefore negatively impact on the development of GVHD and GVL.

Thus, we hypothesized that high concentrations of sMICA before and during the early phases of transplantation could be a biomarker to identify patients at risk of developing GVHD. The current literature on the role of sMICA in allogeneic HSCT mainly concerns MICA polymorphisms (6, 8, 11, 15–18), while one group reported that elevated sMICA concentrations at day 100 post-transplantation were associated with the development of chronic GVHD (16). We specifically hypothesized that sMICA may act as an acute phase reactant that increases during the engraftment phase and investigated possible associations with the development of acute and/or chronic GVHD.

## Methods

### Design

This was a prospective, single center, non-interventional study. This study complied with the principles set forth in the Declaration of Helsinki. The Ethics Committee of the Medical University of Vienna approved the study before its initiation (EK number 1483/2018).

### Patients

We included 48 adult patients with malignant and non-malignant hematological diseases undergoing allogeneic HSCT at the Bone Marrow Transplantation unit of the Department of Internal Medicine I at the Medical University of Vienna, who were eligible for this study between 2020 and 2021. Follow up was 1 year for each patient starting with HSCT (=day 0).

Details of the patient eligibility criteria and HLA-typing are reported in Supplementary material.

### Diagnosis of GVHD

Diagnosis of GVHD was established by regular physical examinations and routine laboratory examinations in the in- or outpatient setting after HSCT. Biopsies were performed if deemed necessary for establishing correct differential diagnosis by treating physicians. Acute GVHD was defined and graded according to the MAGIC criteria (19); chronic GVHD according to the NIH criteria (20).

### Sampling, processing, and storage of samples

Blood samples were drawn at baseline before conditioning chemotherapy (PRE), during the peri-engraftment phase approximately every other day (E, E1-E4) starting on the first day of neutrophil counts >0.5G/L, and at day 100 after HSCT (E100). Engraftment was expected between day 14 to 28 after CD34^+^-cell infusion (=day 0) as defined in the consensus paper from the American Society for Transplantation and Cellular Therapy (ASTCT) as the first of three consecutive days with cell counts of neutrophils >0.5 G/L and platelets >20 G/L in the absence of platelet transfusions for 7 consecutive days (21). Blood samples were centrifuged at 2500 g for 15 min at 4 degrees Celsius within 1 h of sampling. Plasma and serum were transferred in respective 1.5 mL tubes and stored for later analysis at −80 degrees Celsius. Clinical events and patient-specific medication were documented in the medical history of each patient and obtained by electronic chart review. Differential blood counts, blood chemistry (e.g., electrolytes, liver and kidney function parameters, etc.), and immunologic parameters [i.e., C-reactive protein (CRP)] were measured by the central laboratory of the General Hospital of Vienna on a daily routine basis at least until engraftment. Von Willebrand Factor (VWF) and sMICA were measured by commercially available enzyme-linked immunosorbent assays (ELISA) according to the manufacturer’s instructions at the Medical University of Vienna and the University of Graz, respectively (REAADS vWF Antigen ELISA, K034-001 kits, DiaPharma, West Chester, OH, USA; Human MICA DuoSet ELISA, DY1300 kits, R&D Systems, Minneapolis, MN, USA). CRP and VWF were measured as established parameters of systemic inflammation (22) and endothelial cell activation (23).

### Sample size

In patients before transplantation sMICA concentrations were on average 53 pg/mL (95% CI 43–63 pg/mL) and increased to 72 pg/mL (95% CI 61–83 pg/mL) after transplantation (16). The risk of developing chronic GVHD within 3 years after transplantation increased from 46 to 82% when levels exceeded 80 pg/mL, which was the 70th percentile (1) (16). However, currently no information is available for sMICA levels during the peri-engraftment period. Hence, a formal sample size calculation was not possible for this exploratory study, but given these published data (16), we assumed mean sMICA concentrations in patients who will not develop GVHD of 50 pg/mL and a mean of 100 pg/mL in patients who will develop GVHD during engraftment with a standard deviation of 50 pg/mL in each group, with an alpha error of 5% and a power of 80%. A sample size of 36 patients would be sufficient to show a statistically significant difference between groups based on these assumptions (Wilcoxon-Mann–Whitney test for two groups, G. Power 3.1 Version 3.1.9.6). To be more conservative and to account for potential dropouts we increased the sample size to 48.

#### Statistical analysis

The primary endpoint of this study was to compare sMICA concentrations between patients who developed acute or chronic GVHD and those who did not. We applied a hierarchical testing procedure. At first, we calculated a repeated measures analysis of variance (ANOVA) to compare sMICA concentrations between patients with or without any form of GVHD including all available time points. If a significant difference was found, pairwise comparisons of all timepoints were conducted between groups in an exploratory manner using the non-parametric Mann–Whitney-U Test. We repeated the overall analysis excluding the day 100 value for any form of GVHD, because it lacks relevance for acute GVHD. Further exploratory analyses were conducted for the two subgroups (i) acute GVHD and (ii) chronic GVHD. We excluded all patients who suffered from chronic GVHD from the (i) acute GVHD subgroup, and all patients who suffered from acute GVHD from the (ii) chronic GVHD subgroup. The kinetics of other biomarkers (i.e., CRP and VWF) were analyzed similarly to sMICA. To assess changes over time of a single biomarker a Friedman ANOVA was conducted. To investigate the diagnostic performance of the biomarker we performed receiver operating characteristic (ROC) curve analyses. Furthermore, univariate and multivariate logistic regression models were calculated to investigate effect sizes. We calculated multivariate logistic regression models using the “backward elimination” procedure (Wald test). First, all covariates of interest were included into the model. However, given the small sample size that may limit the number of covariates for such a model (rule of thumb: one covariate for 10 patients), we also conducted multiple multivariate models including only two covariates each (sMICA and a second parameter) using the “enter” procedure and investigated whether these covariates affected the obtained results.

Correlations were calculated using the non-parametric Spearman test. We analyzed associations of sMICA with mortality or relapse at day 100 or at the 1-year follow up visit.

This was an exploratory study, therefore no correction for multiple testing was applied. However, we aimed to reduce the number of statistical tests by implementing a hierarchical test procedure.

In case of missing values, a last observation carried forward approach was conducted for the repeated measures ANOVA. All tests were 2-sided, with type I error rate fixed at 0.05. Statistical analyses were performed with SPSS 29 software (IBM® SPSS®, NY, USA). Figures were created with GraphPad Prism 10 (Boston, MA, USA).

## Results

### Patient characteristics

Between 2020 and 2021 a total of 48 patients were enrolled into this study. Patients who failed to successfully engraft (*n* = 2) or who died within the first week of HSCT without the possibility of developing GVHD (*n* = 2) were excluded from the final analysis. Therefore, we ultimately included the remaining 44 patients in our analysis. Baseline characteristics, including demographics, underlying disease and transplantation characteristics are reported in Table 1.

**Table 1:** Patient, donor, disease, and transplantation characteristics.

Characteristics	Values
Patients (*n* = 44)	
Median age, y (IQR 1–3)	54 (43–61)
Male, *n* (%)	29 (66)
Female, *n* (%)	15 (34)
ECOG at time of transplantation, *n* (%)	
0	42 (96)
1	1 (2)
1–2	1 (2)
Median days in hospital, *n* (IQR 1–3)	28 (26–33)
Patient-donor sex matched, *n* (%)	24 (55)
Male donor to female recipient, *n* (%)	9 (20)
Female donor to male recipient, *n* (%)	11 (25)
Patient–donor serological status for cytomegalovirus, *n* (%)	
Matched serological status (seropositive-seropositive, seronegative-seronegative)	33 (75)
Seropositive-seronegative	6 (14)
Seronegative-seropositive	5 (11)
Patient–donor AB0 blood group mismatches (minor, major and bi-directional), *n* (%)	26 (59)
Underlying Disease, *n* (%)	
ALL	3 (7)
AML	27 (61)
CLL	1 (2)
CML	1 (2)
CMML	2 (5)
B-NHL	3 (7)
MDS	4 (9)
MF	1 (2)
SAA	2 (5)
Disease stage at transplantation, *n* (%)	
CR1	24 (55)
CR2	5 (11)
CR3	3 (7)
Active disease	11 (25)
Unknown	1 (2)
Transplantation	
Source of stem cells, *n* (%)	
PBSC	44 (100)
BM	1 (2)
Median CD34 + cell count, *n* (IQR 1–3)	6.64 (4.97–7.80)
Donor matching, *n* (%)	
MSD (10/10)	11 (25)
MUD (10/10)	25 (57)
HAPLO (5/10)	8 (18)
Conditioning regimens, *n* (%)	
MAC	1 (30)
Flu/TBI12	3
TB3F	10
TRMAC/RIC	27 (61)
Flu/Bu4	12
Flu/TBI8	3
Flu/Treo30	8
TB2F	4
NMA	4 (9)
Flu/Cy	2
Flu/Cy50/TBI2	2
GVHD prophylaxis, *n* (%)	
CSA + MMF + ATG	36 (82)
Baltimore PTCy + CSA + MMF	8 (18)

ALL, acute lymphoblastic leukemia; AML, acute myeloid leukemia; ATG, anti-thymocyte globulin; BM, bone marrow; B-NHL, B-Non Hodgkin lymphoma; Bu, Busulfan; CLL, chronic lymphocytic leukemia; CMML, chronic myelomonocytic leukemia; CMV, cytomegalovirus; CR1,2,3, complete remission 1,2,3; CSA, cyclosporin A; CML, chronic myeloid leukemia; Cy, cyclophosphamide; ECOG, Eastern Cooperative Oncology Group; Flu, Fludarabine; GVHD, graft-versus-host disease; HAPLO, haploidentical donor; IQR, interquartile range; MAC, myeloablative conditioning; MDS, myelodysplastic syndrome; MF, myelofibrosis; MMF, mycophenolate mofetil; MUD, matched unrelated donor; NMA, non-myeloablative conditioning; PBSC, peripheral blood stem cells; PTCy, post-transplantation cyclophosphamide; RIC, reduced intensity conditioning; SAA, severe aplastic anemia; sAML, secondary acute myeloid leukemia; TBI, total body irradiation; Treo Treosulfan; TRMAC, toxicity-reduced myeloablative conditioning.

### Conditioning and GVHD prophylaxis

Most transplantations were performed with reduced-toxicity myeloablative (RTMAC) and reduced-intensity (RIC) conditioning regimens (61%).

Patients received GVHD prophylaxis with Cyclosporine A (CSA) and mycophenolate mofetil (MMF) in combination with antithymocyte globulin (ATG) or post-transplantation cyclophosphamide (PTCy).

### Engraftment

Time to leukocyte engraftment was 14 days (IQR 1–3: 12–18 days), and time to platelet engraftment was 12 days (IQR 1–3: 10–14 days).

### GVHD

**Figure 1:**
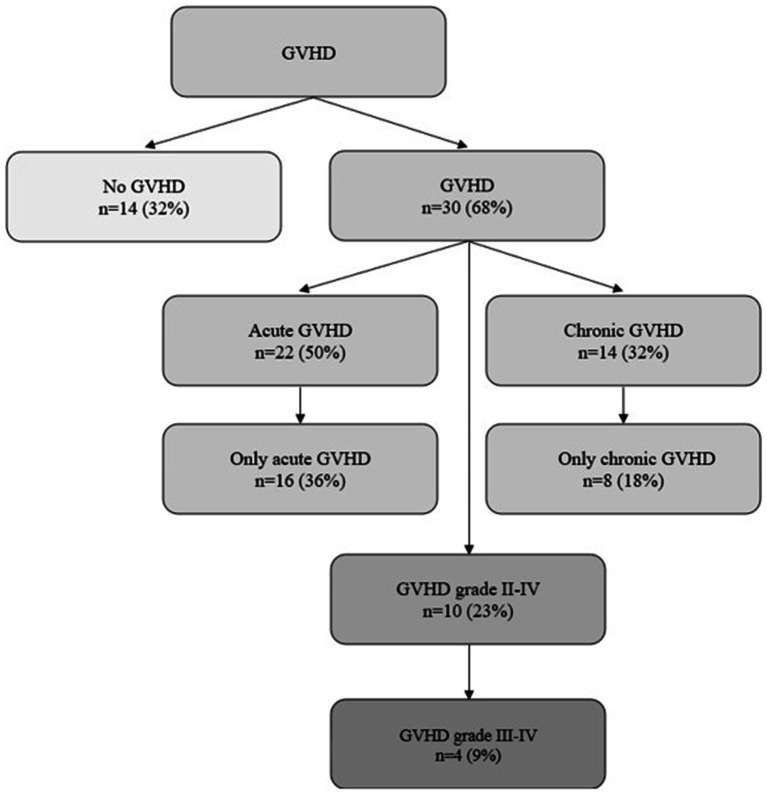
GVHD characterization.

In our population, 30 (68%) patients developed GVHD (i.e., acute and/or chronic) during the follow-up period: 16 (36%) acute GVHD, 8 (18%) chronic GVHD, 6 (14%) acute and chronic GVHD. Ten (23%) patients suffered from significant (grade II-IV) GVHD, of whom 4 (9%) patients developed severe to life-threatening (grade III-IV) GVHD. Characterization of GVHD and organ specific grading of GVHD is shown in Figure 1 and Table 2, respectively.

**Table 2:** Organ specific grading of GVHD.

Acute GVHD	
Skin	86%
Gastrointestinal tract	9%
Liver	5%
Oral mucosa	5%
Eyes	5%
Lungs	5%
Chronic GVHD	
Skin	57%
Liver	29%
Oral mucosa	7%
Eyes	21%

### Survival and relapse

Forty-two patients (95%) survived until day 100, 37 (84%) survived the follow-up period until day 365, and 2 (5%) patients were lost to follow-up after day 100 (Supplementary Figure 1).

During the follow-up period, a total of 6 (14%) patients relapsed on days 62, 136, 142, 188, 260, and 384, respectively.

### sMICA

The concentrations of sMICA changed over time in overall statistical testing (*p* = 0.041).

Soluble MICA concentrations did not differ significantly at the time-points pre-transplantation and during engraftment, although they decreased at day 100 compared with the peri-engraftment time-points E2 and E3 (*p* = 0.011 and *p* = 0.031, respectively).

C-reactive protein concentrations and VWF concentrations increased significantly during the engraftment period (*p* < 0.001 for both parameters). However, these parameters did not significantly differ between patients with or without acute or chronic GVHD. Furthermore, there was no significant correlation between sMICA and CRP or VWF.

**Figure 2:**
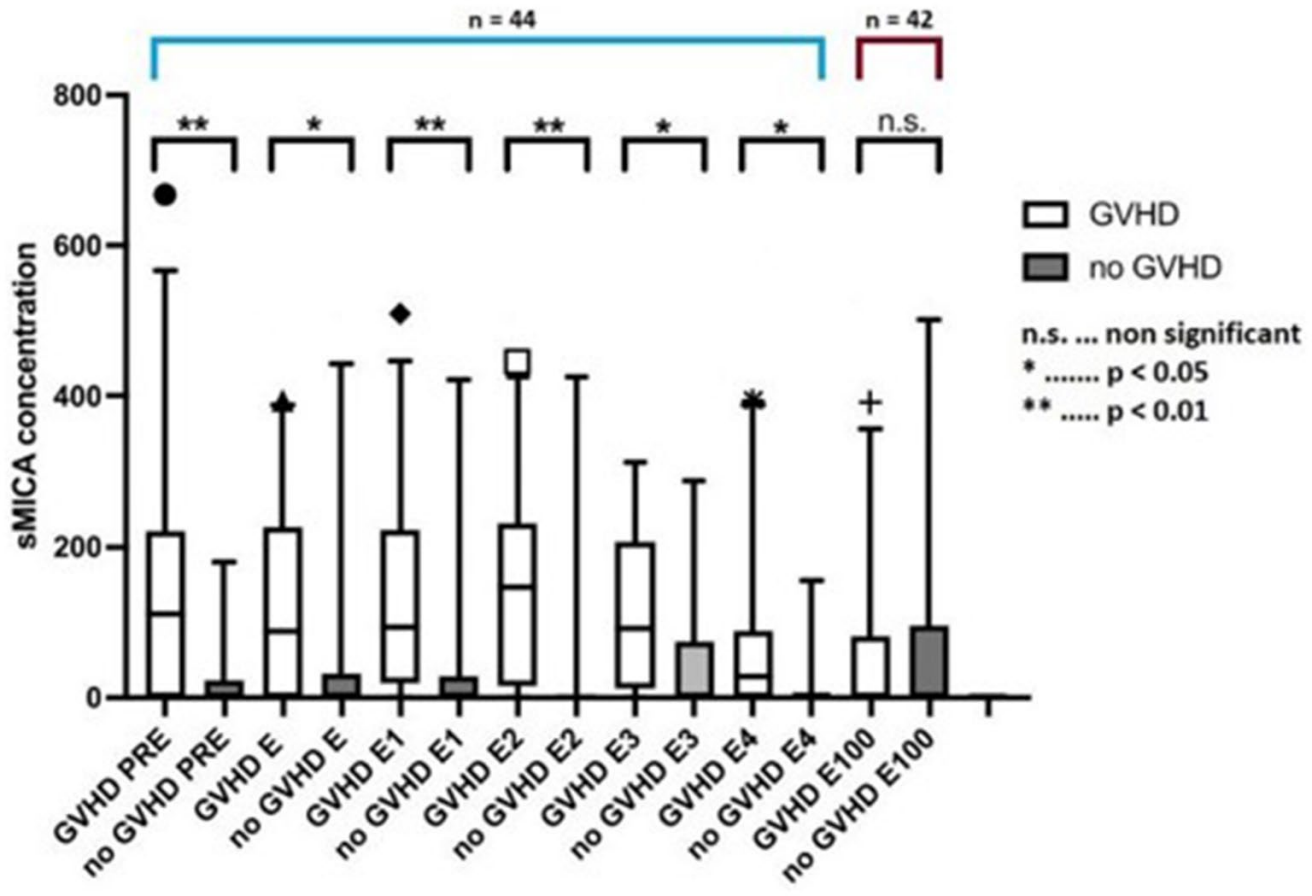
sMICA concentrations between pre-transplantation and day 100 in patients without GVHD and with GVHD. sMICA soluble MHC class I chain-related protein A, GVHD graft-versus-host disease, PRE time-point before conditioning regimen, E time-point where leukocyte counts were >500/μL for the first time after aplasia, E1-4 time-points following E in every-other-day-intervals, E100 day 100 after transplantation.

**Figure 3:**
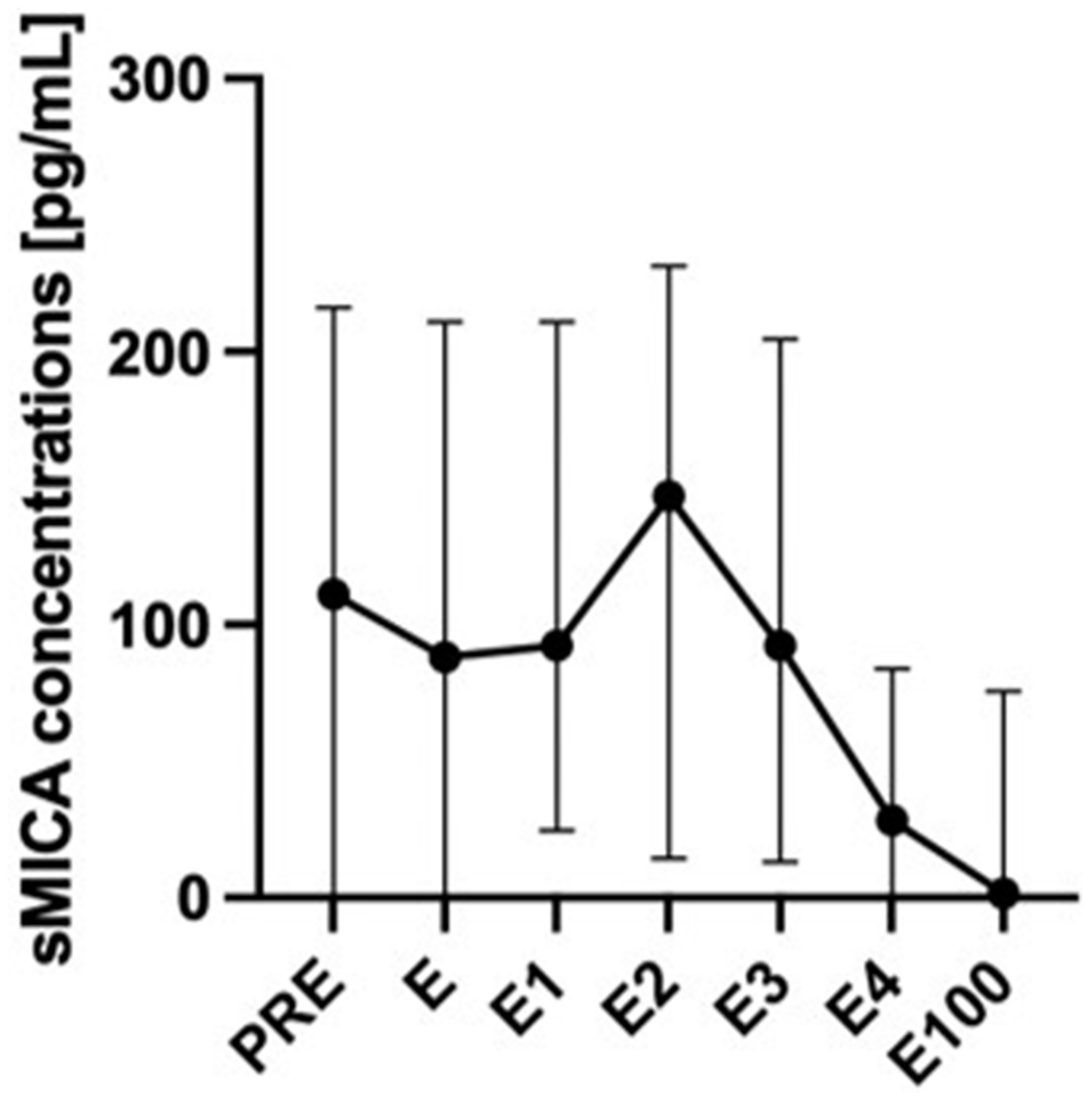
sMICA concentrations over time in patients with acute or chronic GVHD. sMICA soluble MHC class I chain-related protein A, GVHD graft-versus-host disease, PRE time-point before conditioning regimen, E time-point where leukocyte counts were >500/μL for the first time after aplasia, E1-4 time-points following E in every-other-day-intervals, E100 day 100 after transplantation.

Soluble MICA concentrations were significantly higher in patients who developed acute or chronic GVHD compared with patients who did not develop GVHD (Figure 2; *p* = 0.039 including day 100 value, *p* = 0.017 excluding day 100 value). In pairwise comparisons, sMICA concentrations differed between groups until the end of engraftment. Interestingly, at day 100 this difference diminished possibly due to overall diminishing sMICA concentrations. The time-course of sMICA concentrations in patients who developed acute or chronic GVHD is shown in Figure 3.

The median of sMICA concentrations at the pre-transplant timepoint was 33.5 pg/mL. Half of the patients with sMICA concentrations below the median developed any form (acute, chronic, or both) of GVHD (50%, 11 of 22), while 86% of patients with sMICA concentrations above 33.5 pg/mL developed any form of GHVD (19 of 22). Overall, 16 patients developed acute GVHD only. Seven of 22 patients with sMICA concentrations below the median developed acute GVHD, while this was true for nine of 22 patients (41%), who had sMICA concentrations of >33.5 pg/mL. In total, 8 patients developed chronic GVHD only. Two of 22 patients (9%) with sMICA concentrations below the median and six of 22 (27%) patients with sMICA concentrations above the median developed chronic GVHD. Six patients developed both acute and chronic GVHD. Two of 22 patients (9%) with sMICA concentrations below the median and four of 22 patients (18%) with sMICA concentrations above 33.5 pg/mL developed both acute and chronic GVHD.

**Figure 4:**
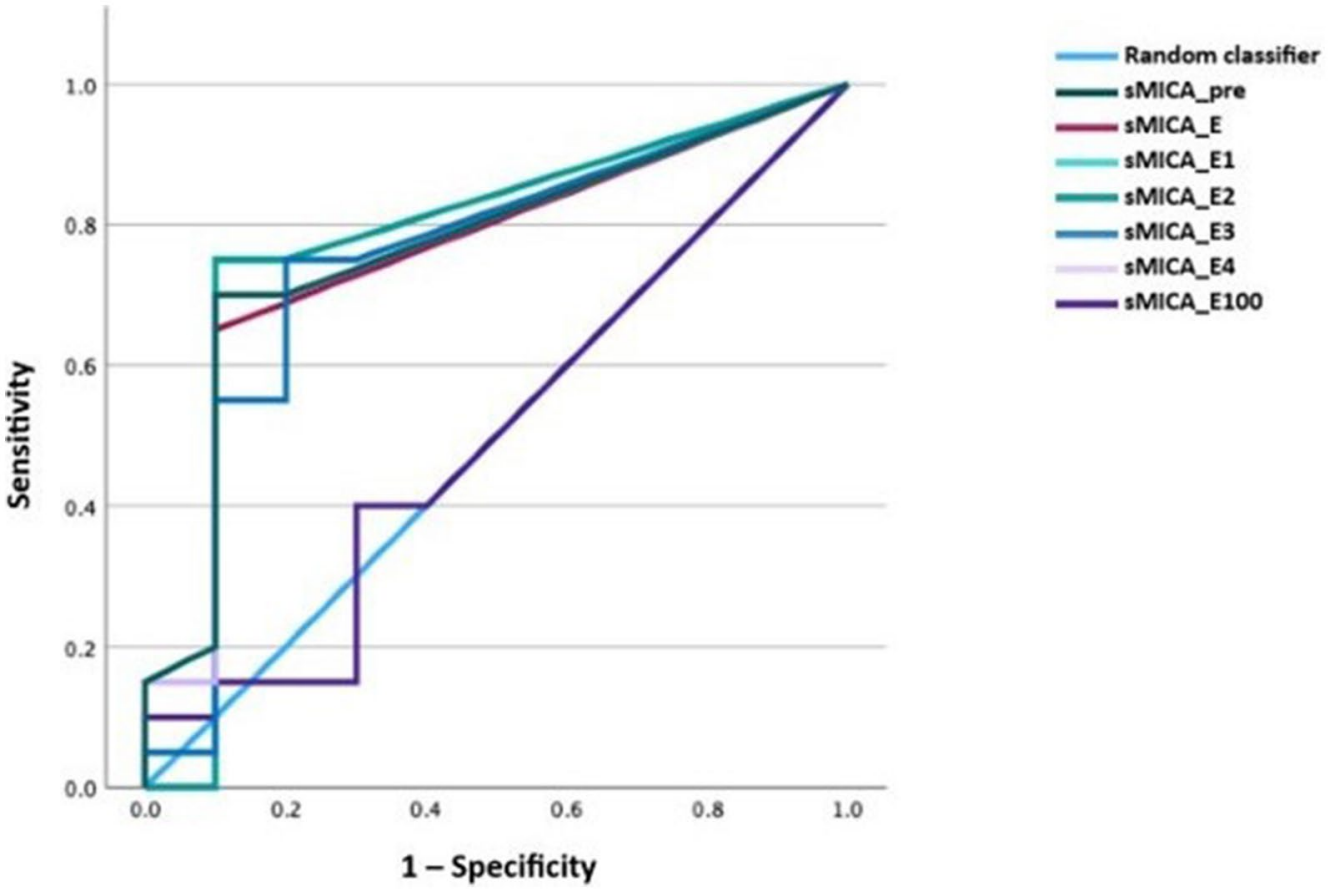
ROC curve of sMICA concentrations at the time-points of interest for acute or chronic GVHD.

**Figure 5:**
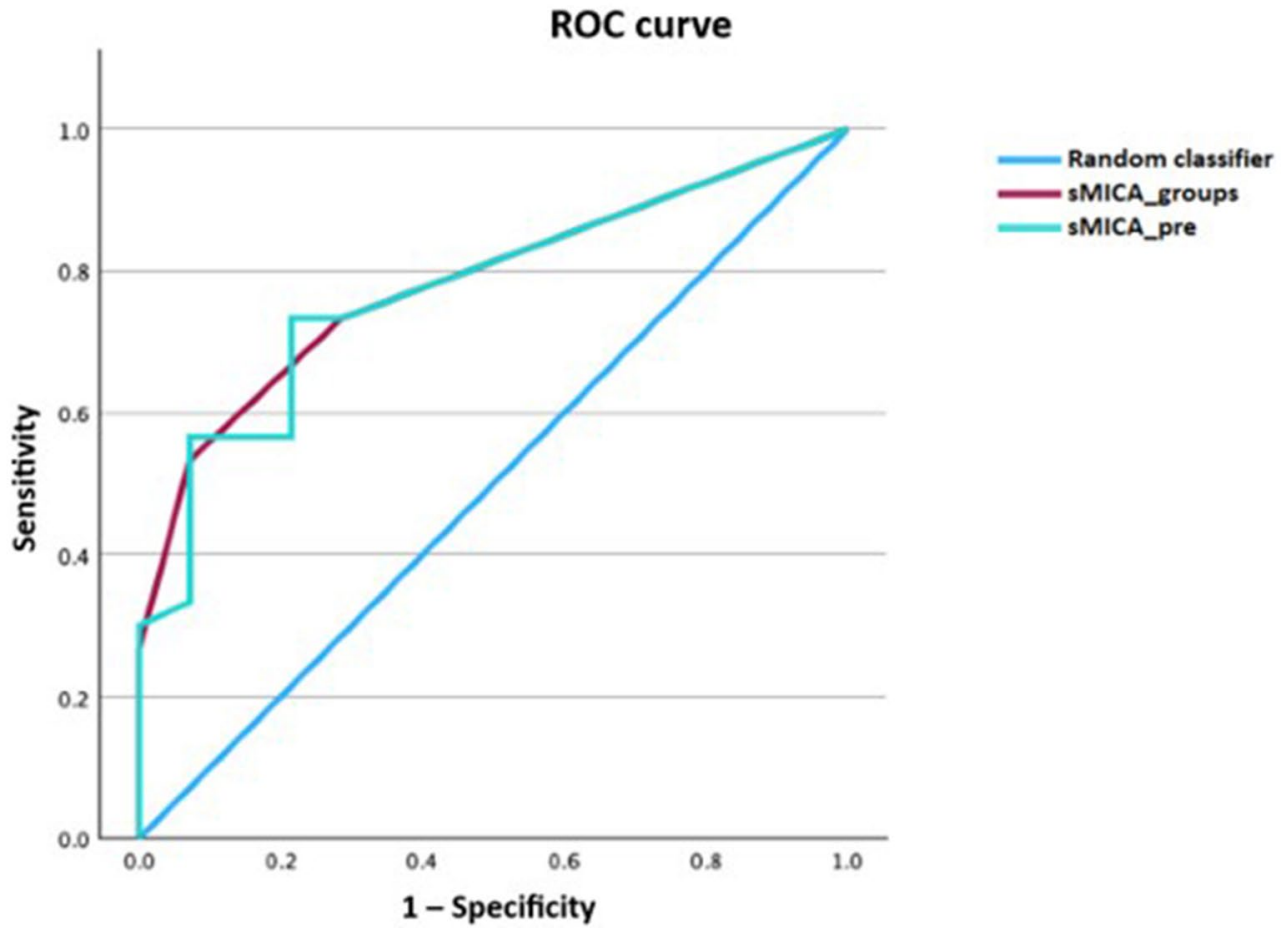
ROC curve comparison between pre-transplantation sMICA concentrations versus categorized sMICA concentrations (based on the baseline sMICA concentrations) for acute or chronic GVHD. sMICA soluble MHC class I chain-related protein A, GVHD graft-versus-host disease, pre time-point before conditioning regimen, groups patients categorized into four groups based on the baseline sMICA concentration.

We performed receiver-operating characteristic (ROC) curve analysis using the different time-points for the diagnosis of any form of GVHD. The diagnostic performance was comparable for all time-points except for day 100, which was indifferent from the random classifier. The areas under the curve (AUCs) ranged between 0.74–0.78 (*p* < 0.05; Figure 4). Because the pre-transplantation and peri-engraftment sMICA concentrations yielded similar results, we continued our analysis using the earliest time-point (i.e., pre-transplantation). Furthermore, we categorized patients based on the baseline sMICA concentrations into four groups: group 0 with sMICA concentrations of 0 pg/mL (*n* = 18), group 1 with sMICA concentrations of 1–93.5 pg/mL (*n* = 9), group 2 with sMICA concentrations of 94–193 pg/mL (*n* = 9), group 3 with sMICA concentrations of >194 pg/mL (*n* = 8). We repeated the ROC analysis using this categorical variable and compared it with the pre-transplantation concentrations (Figure 5).

The performance of the pre-transplantation sMICA concentrations and the categorized sMICA concentrations was similar with an AUC of 0.78 (*p* < 0.001; 95% CI 0.64–0.91 for the metric variable and 0.65–0.92 for the categorical variable). Soluble MICA concentrations above 93.5 pg/mL had a specificity of 93% for the diagnosis of GVHD, while the sensitivity was only 47%.

We then calculated a binary logistic regression model for the endpoint of GVHD for the metric variable sMICA pre-transplantation. In this model sMICA concentrations increased the risk of developing GVHD with an odds ratio (OR) of 1.014 per 1 pg/mL of sMICA (95% CI 1.002–1.026, *p* = 0.023). We repeated this model with the categorical variable of sMICA as described above. Each category increased the risk of having GVHD with an OR of 3.53 (95% CI 1.43–8.64, *p* = 0.006).

We calculated a multivariate logistic regression model adjusted for the covariates age, sex, administered CD34^+^-cell count, donor type, conditioning regimen, disease, and disease status. However, all those factors were eliminated from the model and only sMICA concentrations remained significant. This analysis may be limited by the small sample size. Thus, we performed sensitivity analyses with two covariates each; (i) sMICA concentrations and (ii) the above-mentioned covariates (using the “enter procedure”). Adjustment had little to no impact on the OR of sMICA on GVHD.

Finally, sMICA concentrations did not show any association with relapse and overall survival (OS) at day 100 or 1 year.

#### Patients with only acute GVHD (*n* = 16)

**Figure 6:**
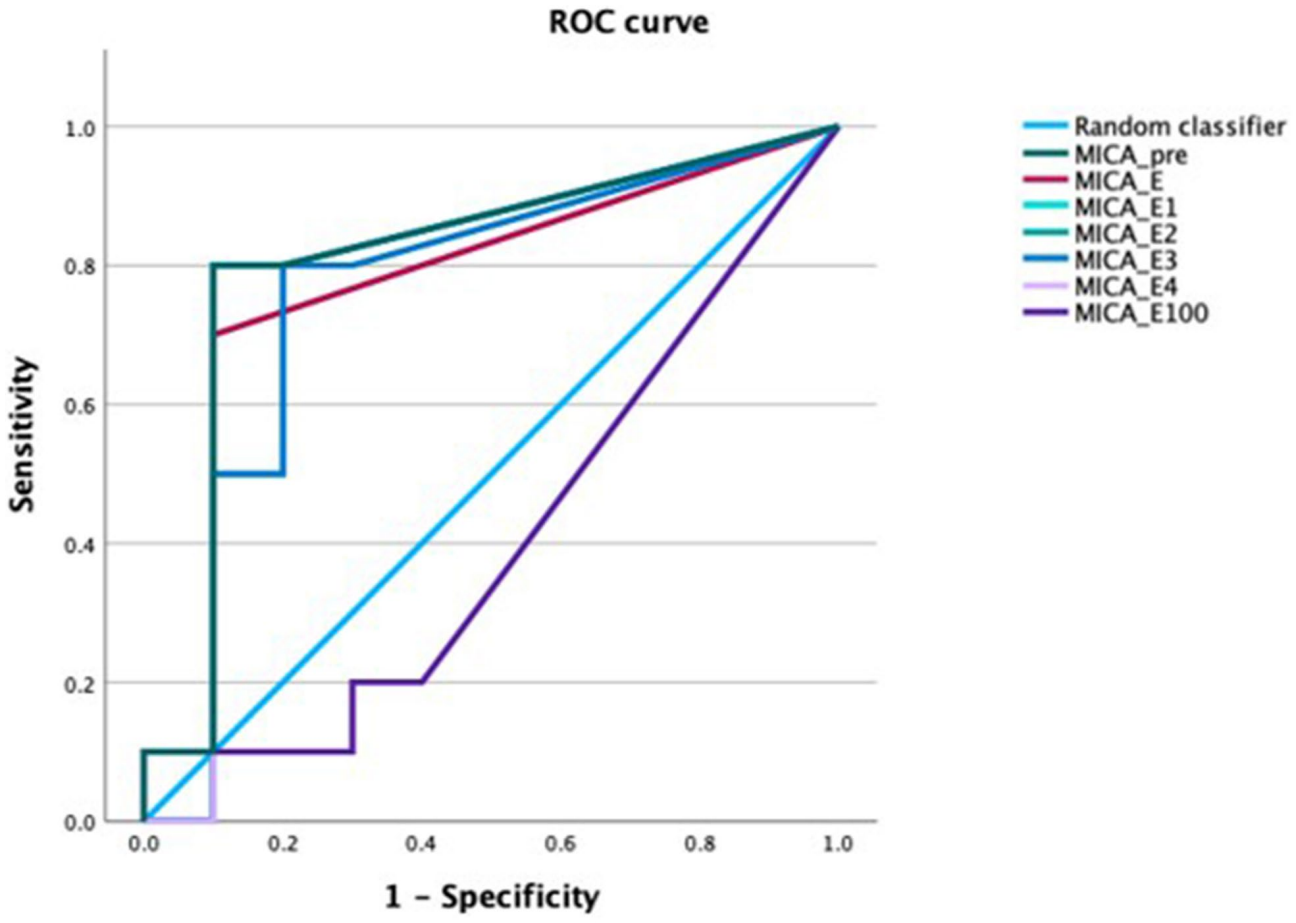
ROC curve of sMICA concentrations at the time-points of interest for the diagnosis of acute GVHD. sMICA soluble MHC class I chain-related protein A, GVHD graft-versus-host disease, PRE time-point before conditioning regimen, E time-point where leukocyte counts were >500/μL for the first time after aplasia, E1-4 time-points following E in every-other-day-intervals, E100 day 100 after transplantation.

In patients with acute GVHD sMICA concentrations were higher compared with patients who did not develop GVHD at almost all time-points. The obvious difference in sMICA concentrations disappeared at day 100. We performed ROC analysis for the diagnosis of acute GVHD. All time-points except for day 100 showed a significantly better diagnostic performance than the random classifier. The AUCs ranged between 0.76 and 0.81. Pre-transplantation sMICA concentrations exhibited the most notable numerical performance. The AUC was 0.76 (95% CI 0.58–0.94, *p* = 0.04). Concentrations of sMICA above 91 pg/mL had a specificity of 93% while the sensitivity was 44% (Figure 6).

Due to the small sample size, we refrained from performing additional analyses.

#### Patients with only chronic GVHD (*n* = 8)

In patients with chronic GVHD sMICA concentrations were numerically higher compared with patients who did not develop any form of GVHD at almost all time-points. The obvious difference in sMICA concentrations disappeared at day 100. Pairwise comparisons did not reveal a statistically significant difference between groups, which is most likely caused by the small sample size of patients with chronic GVHD only.

Due to the small sample size, we refrained from performing additional analyses.

## Discussion

In this study elevated sMICA concentrations in the pre-transplantation and peri-engraftment period after allogeneic HSCT were associated with the occurrence of acute or chronic GVHD with a high specificity, but only with a modest sensitivity. Thus, our results may independently confirm the findings by Boukouaci et al. (16) and even extend them. In our population high sMICA concentrations were associated with acute GVHD, while we could only show a numerical association of elevated sMICA concentrations with chronic GVHD, most probably due to the small number of affected patients (*n* = 8). Of note, for this analysis we excluded all patients who had both, acute and chronic GVHD. Furthermore, the follow-up period of 1-year may have been too short to detect all cases of chronic GVHD.

The main hypothesis of this study was that increased MICA expression contributes to the development of GVHD, because chronic engagement of NKG2D signaling leads to NKG2D endocytosis and degradation. This subsequently causes a reduced responsiveness of NK and T cells to other stimuli (12). This may be advantageous in physiological conditions (e.g., pregnancy), where this mechanism allows for temporarily desirable downregulation of the immune system response to “not-self” antigens (24). Of note, this mechanism is independent of genetic disparities in MICA between donor and host cells which were already shown to trigger alloreactivity with an increased risk of chronic GVHD and poor clinical outcomes (6, 8, 15, 16).

Larghero et al. (25) further corroborate the importance of NK cells by showing that a higher NK cell dose is associated with a lower incidence of chronic GVHD. Interestingly, Salih et al. (26) showed that sMICA concentrations were significantly higher in patients with malignant hematologic diseases compared with healthy volunteers. Thus, one could hypothesize that elevated sMICA concentrations before HSCT may be a sign of residual disease.

Interestingly, in our patient cohort sMICA levels were significantly lower at day 100 than at earlier time-points (i.e., pre-transplantation and peri-engraftment period) in patients who developed acute or chronic GVHD which is in contrast to the published data by Boukouaci et al. (16) Several reasons may explain this finding: first, the decreasing concentrations could be attributed to a lower tumor burden and a success of HSCT; second, the immunosuppressive treatment for acute GVHD; third, the study by Boukouaci et al., published in 2009, used conditioning regimens and supportive and immunosuppressive treatments that have evolved significantly over the last 15 years; fourth, their study primarily used bone marrow as the stem cell source, whereas our cohort used peripheral blood stem cells, except for one patient who received both; and fifth, their follow-up period was 3 years, while ours was limited to 1 year post-transplantation.

Unexpectedly, sMICA concentrations remained unaffected by the aforementioned systemic inflammation and endothelial activation, which clearly argues against it being an acute phase reactant. In addition to the analyses presented in this manuscript, we quantified sMICA concentrations in plasma samples from a human endotoxemia trial in healthy volunteers (27). However, infusion of lipopolysaccharide (LPS; 2 ng/kg bodyweight) did not increase sMICA plasma concentrations in healthy volunteers (unpublished data), whereas pro-inflammatory cytokines and biomarkers of endothelial activation increased significantly (27). This finding was unexpected, because compared with healthy controls sMICA concentrations were increased in patients with bacterial infections (28), coronavirus disease 2019 (COVID-19) (29), and in the synovial fluid of inflamed joints in rheumatoid arthritis patients (30). Furthermore, it has been shown that the expression of MICA (and presumably its soluble form) can be upregulated by genotoxic and cytotoxic stress (e.g., chemotherapy, heat shock, proliferative signals, malignant transformation, infection, oxidative stress, etc.) (10, 11). However, in our cohort, there was no correlation of sMICA concentrations with CRP or VWF. This, however, could be an advantage, because sMICA concentrations seem to be independent of systemic inflammation and hence may not be affected by infections (e.g., bacteremia and sepsis) or other conditions. That said, one must exercise caution in this regard, because the available data are inconsistent.

However, the high specificity of elevated sMICA concentrations for the development of GVHD is remarkable and, if these results are confirmed prospectively in a larger sample, sMICA could indeed serve as a risk factor that triggers an intensified immunosuppressive regimen or, at least, a closer clinical follow up that would allow early detection of GVHD. Of course, this is all subject to further clinical research.

## Limitations

This study presents valuable insights into the biomarker profile of the pre-transplantation, peri-engraftment and post-transplantation period, further contributing to our understanding of allogeneic HSCT. However, it is important to acknowledge certain limitations that warrant consideration when interpreting the results and drawing conclusions.

One of the most relevant limitations of this study is the relatively small size of the patient cohort. The number of patients limits the statistical power of our findings, especially considering the results of sMICA in chronic GVHD.

We did not perform MICA-genotyping and did not measure anti-MICA or anti-sMICA antibodies, which is an obvious limitation for the interpretation of our data. Future studies should consider incorporating such measurements (at least anti-sMICA antibodies) to provide a more holistic understanding of the underlying mechanisms.

This study was a single center study, which might limit the generalizability of the findings to other healthcare settings or regions. Multi-center studies involving different geographic locations could offer a broader perspective on the topic.

Even though efforts have been made to address confounding variables, there remains a chance that factors we have not measured or considered could affect the relationships we are observing. Consideration of these potential confounders could make the study’s conclusions stronger.

Finally, the follow-up period of 1 year per patient was relatively short, especially with regards to the development of chronic GVHD.

## Conclusion

Soluble MICA concentrations before and during the early stages post transplantation were associated with the development of acute and/or chronic GVHD. Its potential use as a biomarker in the prediction of GVHD needs to be confirmed in larger, multi-center studies.

## Data Availability

Data will be made available upon reasonable request to the corresponding author.
