# Supplementary material for: Soluble MHC class I chain-related protein A is a specific biomarker for the early detection of graft-versus-host disease
Source: Front Med (Lausanne). 2025 Jun 5;12:1580452. doi: 10.3389/fmed.2025.1580452 (PMC12176742; doi:10.3389/fmed.2025.1580452)

Supplementary data:

Patient eligibility criteria:

-inclusion criteria:

- Male and female patients undergoing allogeneic HSCT
- Age ≥18
- Signed Informed Consent Form
- Ability to comprehend the nature of the study and its associated risks and benefits

-exclusion criteria:

- Expected life expectancy < 3 months (i.e., patients with severe sepsis or septic shock, treatment at ICU)
- Treatment with drugs that may interfere with the aims of the study (i.e., TNF-alpha inhibitors, IL-6 inhibitors, etc.), as deemed relevant by the investigators
- Comorbidities that may interfere with the aims of the study, as deemed relevant by the investigators

HLA-typing:

HLA-typing was performed with high-resolution next-generation sequencing (NGS).

Genotyping data of HLA-A, HLA-B, HLA-C, HLA-DRB1, HLA-DRB3, HLA-DRB4, HLA-DQB1, HLA-DQA1, and HLA-DPB1 were available for all donors and recipients.

All patient-donor pairs were 10/10 HLA-matched for HLA-A, HLA-B, HLA-C, HLA-DRB1, and HLA-DQB1.

Supplementary Figure 1: Survival

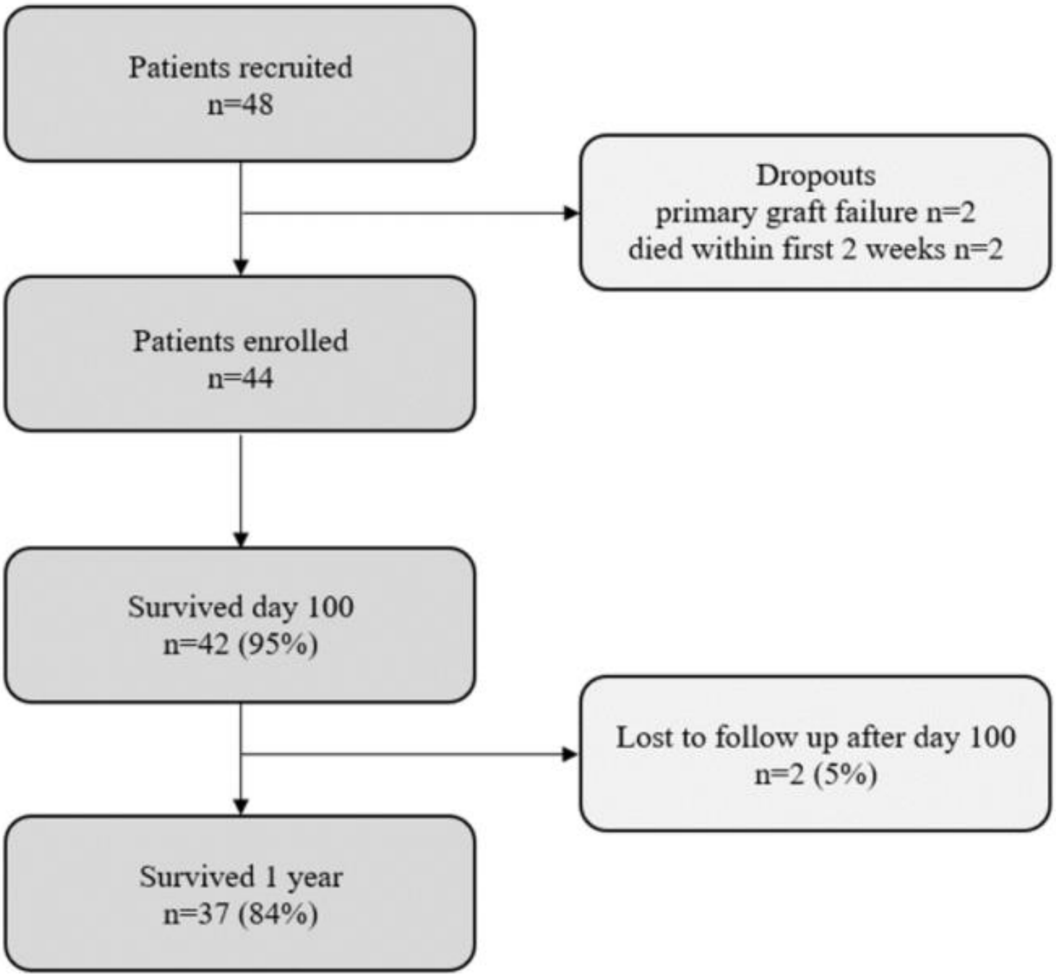

Supplement: Supplementary file 1 [file Data_Sheet_1.pdf]
